# Rapidly Progressive Classic Adamantinoma of the Spine: Case Report and Literature Review

**DOI:** 10.3389/fonc.2022.862243

**Published:** 2022-03-31

**Authors:** Yan Lou, Ying Li, Lei Xu, Xiaoping Jing, Su Chen, Minglei Yang, Hongyu Jiang, Chenglong Zhao, Haifeng Wei, Jianru Xiao

**Affiliations:** ^1^Department of Orthopedic Oncology, Spine Tumor Center, Changzheng Hospital, Naval Military Medical University, Shanghai, China; ^2^Department of Pathology, Changzheng Hospital, Naval Military Medical University, Shanghai, China; ^3^Department of Orthopedic, Chengdu Seventh People’s Hospital, Chengdu, China; ^4^Department of Radiology, Changzheng Hospital, Naval Military Medical University, Shanghai, China; ^5^College of Basic Medical Sciences, Naval Medical University, Shanghai, China

**Keywords:** spinal adamantinoma, en bloc resection, radiotherapy, squamous differentiation, case report

## Abstract

Adamantinoma is a locally aggressive or malignant tumor, accounting for <0.5% of all primary bone tumors. The tumor usually progresses slowly, with a relatively promising prognosis. Primary or metastatic spinal adamantinoma of bone are rarer. Only four cases have been documented till date. We present two cases of aggressive spinal adamantinoma whose microphotography and radiographic appearance were unusual, with extensive involvement of multiple segments and rapid progression. Case 1 was a 36-year-old woman, presenting with back pain, progressive numbness and motor weakness, who was diagnosed with metastatic adamantinoma in the T2, T7, L2, and L4. She underwent spondylectomy three times to resect these lesions, respectively. Case 2 was a 68-year-old male with complaints of severe left back pain. MRI revealed destructive changes in T1-T4. He underwent posterior decompression (T1-T3), partial vertebrectomy (T2), fixation and fusion (C5-C7, T4-T6). The pathology of two patients was metastatic spinal adamantinoma, whose primary lesions were from tibia and femoral adamantinoma, respectively. Rapid squamous progression was observed in specimens of T2 and T7 lesions of Case 1 in two months. Twenty-five months after surgery, Case 1 developed paralysis, but she refused to receive further examination and treatment. Two months after surgery, Case 2 presented with an upper back pain again. The MRI revealed an increase in osseous destruction and paravertebral mass size. He was administered radiotherapy, with his upper back pain partially relieved. The biological behavior of classic adamantinoma is highly unpredictable, often exhibiting more aggressive behavior upon recurrence or metastasis. The pathological diagnosis of adamantinoma should be confirmed by preoperative biopsy. En bloc resection with a wide margin is the preferred treatment for primary spinal adamantinoma. Radiation therapy can partially relieve the pain.

## Introduction

Adamantinoma is a rare locally aggressive or malignant skeletal tumor, accounting for <0.5% of all primary bone tumors (1, 2). It was first described by Maier in 1900 and subsequently reported again by Fischer in 1913, and both scholars noted that the tumor had similar histological features to odontogenic ameloblastoma (2). Subsequently, there were sporadic case reports, but it was not until 1954 that Mario Campanacci officially named the tumor and described its biological behavior in detail. Another important contribution of Mario Campanacci was to distinguish adamantinoma from osteofibrous dysplasia (3), which was a benign, self-limited tumor and was common in children. The origin of adamantinoma has been debated for nearly a century. Fischer supported the hypothesis of congenital epithelial cell implantation (2), while other scholars supported traumatic implantation (4) or articular origin (5, 6). Since then, with the development of electron microscopy, histology, and immunohistochemistry, the possibility of epithelial origin of adamantinoma has been more favorably confirmed. By electron microscopy, we could observe tumor tissues with epithelial features such as basement membranes, microvilli and tonofibrils forming desmosomes. Immunohistochemistry showed positive staining for cytokeratin. The epithelial origin guided the clinical histological identification of adamantinoma from tumors with different histological origins.

Adamantinoma usually progresses slowly, with a relatively promising prognosis. It is insensitive to radiotherapy and chemotherapy. En bloc resection with a wide margin is the main treatment for primary adamantinoma (2). Adamantinoma commonly affects the tibia, particularly the anterior diaphysis and metaphysis, accounting for 85%-90% of the cases (7). Primary or metastatic spinal adamantinoma of bone are rarer, and only four cases have been documented in the previous literature (8–11). Our center describes two cases of metastatic spinal adamantinoma with more aggressive biological behaviors and uncommon radiographic appearances. Both of them had multiple lesions in the spine and rapid disease progression. More importantly, we observed the rapid squamous progression of tumor tissues in microphotography within two months, which is unique. In addition, this is the first study to review the clinical characteristics, treatment and prognosis of associated cases that have been reported in the literature, which could help us gain a better understanding of spinal adamantinoma.

## Case Presentation

### Case 1

**Figure 1:**
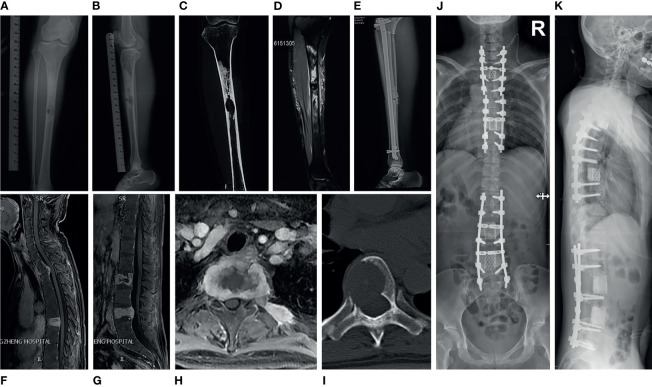
Medical imaging of adamantinoma in case 1. **(A, B)** Anterior and lateral X-ray of the left tibia showed that there were two osteolytic well-circumscribed lesions in the tibial diaphysis, surrounded by a large area of sclerosis. The larger lesion was located in the medullary cavity, the smaller lesion was inside the cortex. **(C)** CT of the left tibia demonstrated an well-demarcated osteolytic lesion in the middle of the diaphysis with surrounding large areas of dense sclerotic bone. **(D)** MRI of the left tibia lesion revealed intense heterogeneous post-contrast enhancement with a central cystic non-enhancing area. **(E)** Lateral radiograph of the post-operative left tibia showed tibial osseous graft reconstruction with placement of an intramedullary rod and interlocking screws. **(F–H)** MRI of spine showed irregular osteolytic destruction in T2, T7, L2, L4 vertebral body and posterior elements, accompanied by the formation of paravertebral soft tissue masses, which demonstrated heterogeneous post-contrast enhancement. **(I)** CT of the thoracic spine showed a round osteolytic destruction with a clear demarcation, which extend into the spinal canal and cause spinal cord compression. **(J, K)** Postoperative X-ray of the spine showed the patient underwent spondylectomy and reconstruction for three times to resect multiple segments tumors.

A 36-year-old woman visited the local clinic in October 2018 with a longstanding history of pain in her left leg (**Supplementary Figure 1**). Radiographs of the left tibia demonstrated two osteolytic well-circumscribed lesions in the diaphysis, surrounded by a substantial area of sclerosis (**Figures 1A**–**C**). Magnetic resonance imaging (MRI) of the left tibia lesions exhibited intense heterogeneous post-contrast enhancement with a central cystic non-enhancing region (**Figure 1D**). Subsequently, she underwent left tibia tumor resection and osseous graft reconstruction with instruments (**Figure 1E**). Her pathologic diagnosis was adamantinoma. Approximately 11 months later, the patient was admitted to our center with a one-month history of back pain, increasing numbness and motor weakness of the left upper and lower extremities. MRI of the spine showed irregular osteolytic destruction in T2, T7, L2, L4 vertebral bodies and posterior elements, accompanied by the formation of paravertebral soft tissue masses. These lesions had T1 hypointense signal, T2 mixed signal, and intense heterogeneous post-contrast enhancement (**Figures 1F**–**H**). Computed tomography (CT) of the thoracic spine exhibited a round osteolytic destruction with a clear demarcation that extended into the spinal canal and compressed the spinal cord (**Figure 1I**). Physical examination indicated hyperreflexia of her bilateral patellar tendon and Achilles tendon reflexes. On September 5, 2019, the patient underwent posterior spondylectomy (T2) in the piecemeal method, fusion with titanium mesh and bone cement, fixation with pedicle screws. Three weeks later, the patient underwent posterior lumbar tumor piecemeal resection (L2, L4), decompression and reconstruction. Two months later, the T7 lesion was excised and reconstructed as well (**Figures 1J, K**). Her back pain and weakness of left limbs significantly improved postoperatively.

**Figure 2:**
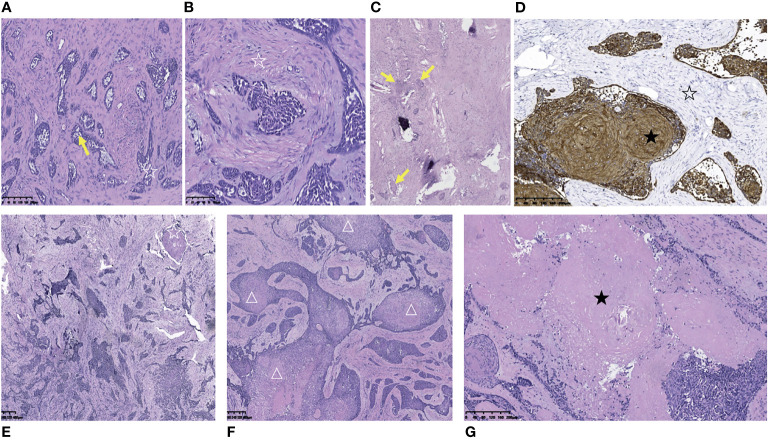
Pathological and immunohistochemical pictures of metastatic thoracic adamantinoma in case 1. **(A)** Microscopically, the tumor tissue was composed of prominent epithelial cell islands and admixed with spindle-shaped fibrous stroma. The basaloid cells in the periphery of the epithelial island were arranged in a palisade pattern (☆), with star-shaped cells (yellow arrow) in the center (Scale 100μ). **(B)** The fibrous stroma was composed of spindle-shaped fibroblasts (☆) with mild cellular atypia with local myxoid degeneration (Scale 100μ). **(C)** Multifocal tumor cell nests (yellow arrow) in bone tissue and hyperplastic fibrous connective tissue (Scale 100μ). **(D)** The tumor cells were diffusely strong positive for AE1/AE3 (★).The stromal cells were negative for AE1/AE3 (☆) (Scale 100μ). **(E)** Microphotography of tumor resection specimen of T2 vertebral body in September 2019. Only a small amount of squamous differentiation could be seen (☆), without any necrotic area (Scale 100μ). **(F, G)** Two months later (November 2019), a large amount of squamous differentiation (△) and multifocal, large areas of necrosis (★) were seen in the resection specimen of the T7 vertebral body tumor (Scale 100μ).

Pathological examination indicated metastatic classic adamantinoma. The gross appearance of the tumor was honeycomb-shaped, gritty, gray-white and grayish-yellow. Microscopically, the tumor was composed of prominent epithelioid cell islands and was admixed with spindle-shaped fibrous stroma. The basaloid cells in the periphery of the epithelial island were arranged in a palisade pattern, with star-shaped cells (yellow arrow) in the center (**Figures 2A, C**). The fibrous stroma was composed of spindle-shaped fibroblasts (☆) with mild cellular atypia with local myxoid degeneration (**Figure 2B**). Immunohistochemically, the fibrous tissue was partially positive for vimentin. The epithelial cells showed coexpression of CK(pan), CK14, CK5/6, epithelial membrane antibody (EMA), P63 and osteonectin (OST) (**Figure 2D**). Interestingly, squamous progression was observed in specimens of different spinal levels in two months. Only a small amount of squamous differentiation could be seen in the T2 lesion (Obtained in September 2019), without any necrotic areas (**Figure 2E**). Two months later, a large amount of squamous differentiation and multifocal, extensive areas of necrosis were seen in the T7 lesion (Obtained in November 2019) (**Figures 2F, G**). In October 2021, the patient developed paralysis, but she refused to receive further examination and treatment.

### Case 2

**Figure 3:**
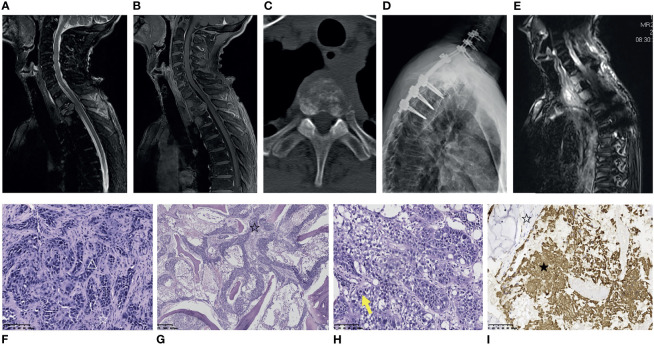
Medical imaging, pathological and immunohistochemical pictures of metastatic thoracic adamantinoma in case 2. **(A, B)** MRI of the thoracic spine demonstrates vertebral body destructive changes from T1-T4 and pathologic fracture of T2 vertebral body. Thoracic lesions were T2-weighted hyperintense signal **(A)**, and post-contrast enhancement **(B)**. **(C)** Thoracic CT shows that the T2 lesion has a mixed lytic/sclerotic appearance with paravertebral soft tissue mass. **(D)** Postoperative X-ray of the spine shows the patient underwent posterior thoracic decompression (T1-T3), partial vertebrectomy (T2), posterior fixation and fusion (C5-C7, T4-T6) with bone cement and pedicle screw. **(E)** Two months after surgery, contrast-enhanced MRI scan reveals an increase in osseous destruction and paravertebral mass size. **(F)** Microscopically, the tumor tissue was composed of prominent epithelial cell islands and admixed with bland spindle cells. Some epithelial islands were tubular cells (△), surrounded by spindle-shaped fibrous stromal cells (☆) (Scale 100μ). **(G)** Microscopically, the epithelial cell islands of the tumor tissue diffusely infiltrated between the trabecular bone in the form of nests (☆), and the trabecular bone was destroyed (Scale 100μ). **(H)** Mitotic phases were uncommon (yellow arrows) (Scale 100μ). **(I)** The tumor cells were positive for AE1/AE3 (★). The stromal cells were negative for AE1/AE3 (☆) (Scale 100μ).

A 68-year-old male patient underwent left femoral tumor resection and reconstruction in the local hospital in September 2019. The postoperative histological diagnosis was adamantinoma (**Supplementary Figure 2**). In July 2021, the patient was admitted to the local clinic with complaints of severe left back pain that radiated to the left neck and upper extremity, requiring oral morphine sustained-release tablets for pain relief. A preoperative MRI and CT of the thoracic spine was performed, which demonstrated severe destructive changes in T1-T4 vertebral bodies, with an associated paravertebral soft tissue mass, and pathologic fracture of the T2 vertebral body. Thoracic lesions had T1 hypointense signal, T2 hyperintense signal, and post-contrast enhancement (**Figures 3A**–**C**). SPECT (single photon emission computed tomography) showed slightly higher tracer uptake in the left femur, T3 vertebral body, and right knee. Two months later, the patient was referred to our center and underwent posterior thoracic decompression (T1-T3), partial vertebrectomy (T2), posterior fixation and fusion (C5-C7, T4-T6) with bone cement and pedicle screws (**Figure 3D**). The patient’s symptoms were relieved postoperatively. The pathology report revealed that the thoracic tumor was classic adamantinoma. The specimen was a gritty mass with gray-white and grayish-brown in color. Microscopically, some epithelial islands were tubular cells, surrounded by spindle-shaped fibrous stromal cells (**Figure 3F**). Some epithelial islands diffusely infiltrated between the trabecular bone in the form of nests (**Figure 3G**). Mitotic phases were uncommon (**Figure 3H**). Immunohistochemical stains indicated the tumor cells were positive for CK(pan), CK5/6, CK19, CK8/18, P53, EMA, P40 (partially positive), P63 (partially positive) and Ki67(70% positive) (**Figure 3I**). Approximately 2 months after the surgery, the patient presented with upper back pain again. The MRI scan revealed an increase in osseous destruction and the size of the paravertebral mass (**Figure 3E**). He was administered radiotherapy at another hospital, with his thoracic back pain partially relieved.

## Discussion

Adamantinoma is an extremely rare, slow-growing malignancy that accounts for <0.5% of primary bone tumors (1, 2). It is more prevalent in adults between the age of 20 to 50 years (12) and rarely affects children (13). About 97% of the reported cases occurred in long tubular bones, primarily in the diaphysis. The most common site was the tibia (80-85%), while less often it was found in the spine (7). To the best of our knowledge, only four cases of primary or metastatic spinal adamantinoma have been reported in the previous literature (8–11) (**Table 1**). Two of them were primary spinal adamantinoma, the other two patients were left tibial adamantinoma metastatic to the spine. The histological origin of adamantinoma has been controversial, and its etiology has not yet been clarified. However, the prevailing view is that the skin basal epithelium is replaced throughout embryonic development, supported by the predominant involvement of the anterior tibia, where the cartilage-formed bone is closest to the skin (14). Additionally, tumor tissues have epithelial properties such as basement membrane, intracytoplasmic hemidesmosomes, tonofilaments, and microfilaments that can be observed by electron microscopy (7, 15).

**Table 1:** Literature Review of Adamantinoma of the Spine.

No.	Author	Primary Site	Age (yr)/Sex	Level	Pathology	Treatment	LR(mo)	Met(mo)	Status atLast Follow-up	Survival(mo)
1	Marteslo JP (2021) (9)	left tibia	38/Female	T7-T10,T12	Adamantinoma	surgery	NR	NR	NR	NR
2	Morales Ciancio RA (2015) (11)	left tibia	45/Male	Sacrum	High-grade dedifferentiated adamantinoma	Surgery, chemotherapy	None	None	Alive	24
3	Nerubay J (1988) (10)	S1	34/Male	S1	Adamantinoma	surgery	6	12	Died of septicemia	18
4	Duan PG (2015) (8)	T11-T12	28/Male	T11-L1	Adamantinoma	surgery	None	None	Alive	24
5	Present case 1	left tibia	36/Female	T2,T7,L2,L4	Classic Adamantinoma	surgery	25	None	Alive	27
6	Present case 2	left femur	68/Male	T1-T3	Classic Adamantinoma	Surgery, radiotherapy	2	None	Alive	4

NR, not reported or clearly specified; LR, Local Recurrence; Met, Metastases.

The initial symptoms in patients with adamantinoma are swelling and pain, which have been reported in 72% of patients (16). Clinical symptoms or radiographic abnormalities can last for more than 30 years before presentation to the hospital (2). Clinically, pathological fracture occurs in 16%-23% of patients (17). In our two cases and previous reports of spinal adamantinoma, the patients often presented with back pain and neurological dysfunction (8, 9, 11). On X-ray, adamantinoma usually appears as an eccentric, occasionally central, lobular lytic lesion with peripheral sclerosis (14). It often has a characteristic soap bubble-like appearance. CT can evaluate the invasion of the cortex, the osteolytic lesion and involvement of the adjacent soft tissues. Adamantinoma appears hypointense on T1-weighted images, hyperintense on T2-weighted images and marked enhancement on post-contrasted MRI images, but these findings are not specific (10). However, the imaging findings of our two patients were not typical. The multifocal, osteolytic tibia lesions of Case 1 were mainly located in the medullary cavity, surrounded by a large area of sclerosis. In Case 1, the thoracic CT showed a purely osteolytic destruction with a distinct demarcation, whereas in Case 2, it revealed a mixed lytic/sclerotic appearance with a paravertebral soft tissue mass.

Adamantinoma is a biphasic tumor characterized by islands of epithelial cells which are surrounded by a relatively bland spindle-cell osteofibrous component. The latest WHO classification divides it into three subtypes: classic adamantinoma, osteofibrous dysplasia-like adamantinoma (OFD-like adamantinoma) and dedifferentiated adamantinoma (18). Osteofibrous-like adamantinoma contains numerous OFD-like areas with small clusters of epithelial cells. In classic adamantinoma, the epithelial component is dominant, with inconspicuous OFD-like regions. In the dedifferentiated adamantinoma, the classic adamantinoma areas gradually transform to a diffuse growing proliferation, where the typical epithelial differentiation is replaced by pleomorphic cells (19, 20). The epithelial part of adamantinoma shows co-expression of vimentin, cytokeratin 5, 14 and 19, as well as EMA. The stromal part reveals immunohistochemical positivity for vimentin. Keratin 8 and 18 are negative in adamantinomas, which are different from many other bone and soft-tissue tumors (7, 14, 21).

Clinically, adamantinoma is often difficult to differentiate from osteofibrous dysplasia. Adamantinoma tends to occur in adults and is characterized by local progressive swelling and intermittent pain, which can involve the medulla and soft tissues. Osteofibrous dysplasia usually occurs in adolescents and presents with a painless mass. Both of them are prone to occur in the tibia and have similar imaging characteristics. The differential diagnosis of adamantinoma and osteofibrous dysplasia mainly relies on pathological and immunohistochemical analysis. In osteofibrous-like adamantinoma, there are small epithelial cell nests, whereas in osteofibrous dysplasia, only a single keratin-positive cell is seen (20).

More importantly, based on the histologic analysis of the tumor tissue samples from different segments of the spine in case 1, we noticed an interesting phenomenon. Histopathology of the T2 vertebral body lesion (resected in September 2019) revealed only a small amount of squamous differentiation without any necrotic area. However, two months later, the T7 vertebral body lesion (resected in November 2019) demonstrated a large amount of squamous differentiation and multifocal, extensive areas of necrosis. The rapid squamous progression of tumor tissues in microphotography within a short period of time suggested that the patient’s tumor was aggressive in nature, which might be consistent with the imaging manifestations of extensive multi-level spinal destruction after primary left tibia adamantinoma resection. Squamous progression indicated that the areas of squamous differentiation in tumor lesions are significantly more extensive than before. Previous studies have reported that lack of squamous differentiation is a risk factor for recurrence or metastasis of adamantinoma (18, 22). However, this case does not seem to be consistent with the literature, more case studies are needed to further validate the findings.

Because adamantinoma is resistant to radiotherapy and chemotherapy, radical resection aimed at obtaining a wide margin is the preferred choice (23). As initial treatment is critical, open or needle biopsy is necessary to avoid curettage (2). In classic adamantinoma, the recurrence rate after intralesional curettage may be as high as 90% (18). In a study of 70 patients with adamantinoma, the 5-year survival rate was at 95%-98.8% (12, 22), while the 10-year survival rate was 87.2% (24). However, the biological behavior of adamantinoma is highly unpredictable, and usually presents with a more aggressive pattern when it relapses (25, 26). In the cohort reported by Schutgens EM et al., 27% (6/28) of patients with recurrent adamantinoma developed local recurrence after more than ten years of follow-up (17). Adamantinoma will metastasize as well, mainly to the lungs and local lymph nodes. Widespread metastases in the spine and rapid progression were the common features of two patients in our series. Although adamantinoma is insensitive to radiotherapy, we found radiation therapy can partially relieve back pain and reduce the dose of opioid analgesics in one of our patients. In addition, it was reported that targeted therapy with tyrosine kinase inhibitors such as sunitinib and pazopanib has achieved some durable responses in some patients (27, 28).

Adamantinoma has some similarities in biological behavior with giant cell tumor of bone and desmoplastic fibroma, exhibiting a locally aggressive nature and unpredictable prognosis. At the imaging level, they usually reveal osteolytic bone destruction. These tumors are not sensitive to radiotherapy and chemotherapy, with a relatively high local recurrence after surgery. Recently, De Vita A, et al. studied bone-vicious-cycle- and neoangiogenesis-related genes expression in giant cell tumor of bone and desmoplastic fibroma, indicating the upregulation of RANK-L, RANK, OPN, CXCR4, RUNX2 and FLT1 and the downregulation of OPG and CXCL12 genes. In particular, the axis RANK/RANKL/OPG was significantly unbalanced towards the bone resorption activation, which is responsible for orchestrating the bone vicious cycle and the activation of osteoclastogenesis potential. Furthermore, *in vitro* and *in vivo* zebrafish analyses provided evidence for suggesting the combination of denosumab and multitarget TKI inhibitor lenvatinib as a promising therapeutic strategy in GCTB and DF compared to monoregimen chemotherapy (29). This report provided useful methods and ideas for the study of the molecular mechanism and potential therapeutic targets in adamantinoma.

The main limitations of our analysis are the observational nature and retrospective design, which implies the risk of patient selection bias. Spinal adamantinoma is very rare and our sample size is small. In this study, we mainly described the clinical features, imaging and histological features, treatment and outcomes of patients, but statistical analysis of the prognostic factors cannot be performed. In the future, multicenter, prospective, large-scale studies are warranted to evaluate potential prognostic factors.

## Conclusion

Although the classic adamantinoma is considered as a locally aggressive or malignant tumor that progresses slowly, 12%–29% of patients will develop metastases (mostly in the lungs) during long-term follow-up. Positive resection margins are risk factors for local recurrence and distant metastasis. Moreover, the biological behavior of adamantinoma is highly unpredictable, often exhibiting more aggressive behavior upon recurrence or metastasis. The pathological diagnosis of adamantinoma should be confirmed by preoperative biopsy. En bloc resection with a wide margin is the preferred treatment for primary spinal adamantinoma. Despite the fact that adamantinoma is resistant to radiotherapy and chemotherapy, we discovered that radiation therapy can partially relieve the pain.

## Data Availability Statement

The original contributions presented in the study are included in the article/**Supplementary Material**. Further inquiries can be directed to the corresponding authors.

## Ethics Statement

The studies involving human participants were reviewed and approved by The ethics committee of Shanghai Changzheng Hospital. The patients/participants provided their written informed consent to participate in this study.

## Author Contributions

YaL, HW and JX designed the case report. LYa, MY, and CZ participated in the operation and management of the patients. YiL, JX, and HJ prepared radiological and histology figures and provided immunohistochemical analysis. YaL, LX, SC, and HJ reviewed the literature and drafted the article. YaL, YiL and LX contributed equally to this work and all should be considered as first author. All authors contributed to the article and approved the submitted version.

## Funding

We gratefully acknowledge the financial support by a grant, Shanghai Sailing Program (21YF1457100).

## Conflict of Interest

The authors declare that the research was conducted in the absence of any commercial or financial relationships that could be construed as a potential conflict of interest.

## Publisher’s Note

All claims expressed in this article are solely those of the authors and do not necessarily represent those of their affiliated organizations, or those of the publisher, the editors and the reviewers. Any product that may be evaluated in this article, or claim that may be made by its manufacturer, is not guaranteed or endorsed by the publisher.
