# Supplementary material for: Rapidly Progressive Classic Adamantinoma of the Spine: Case Report and Literature Review
Source: Front Oncol. 2022 Mar 31;12:862243. doi: 10.3389/fonc.2022.862243 (PMC9008729; doi:10.3389/fonc.2022.862243)
Supplement: Supplementary file 1 [file Presentation_1.pptx]

## Slide 1
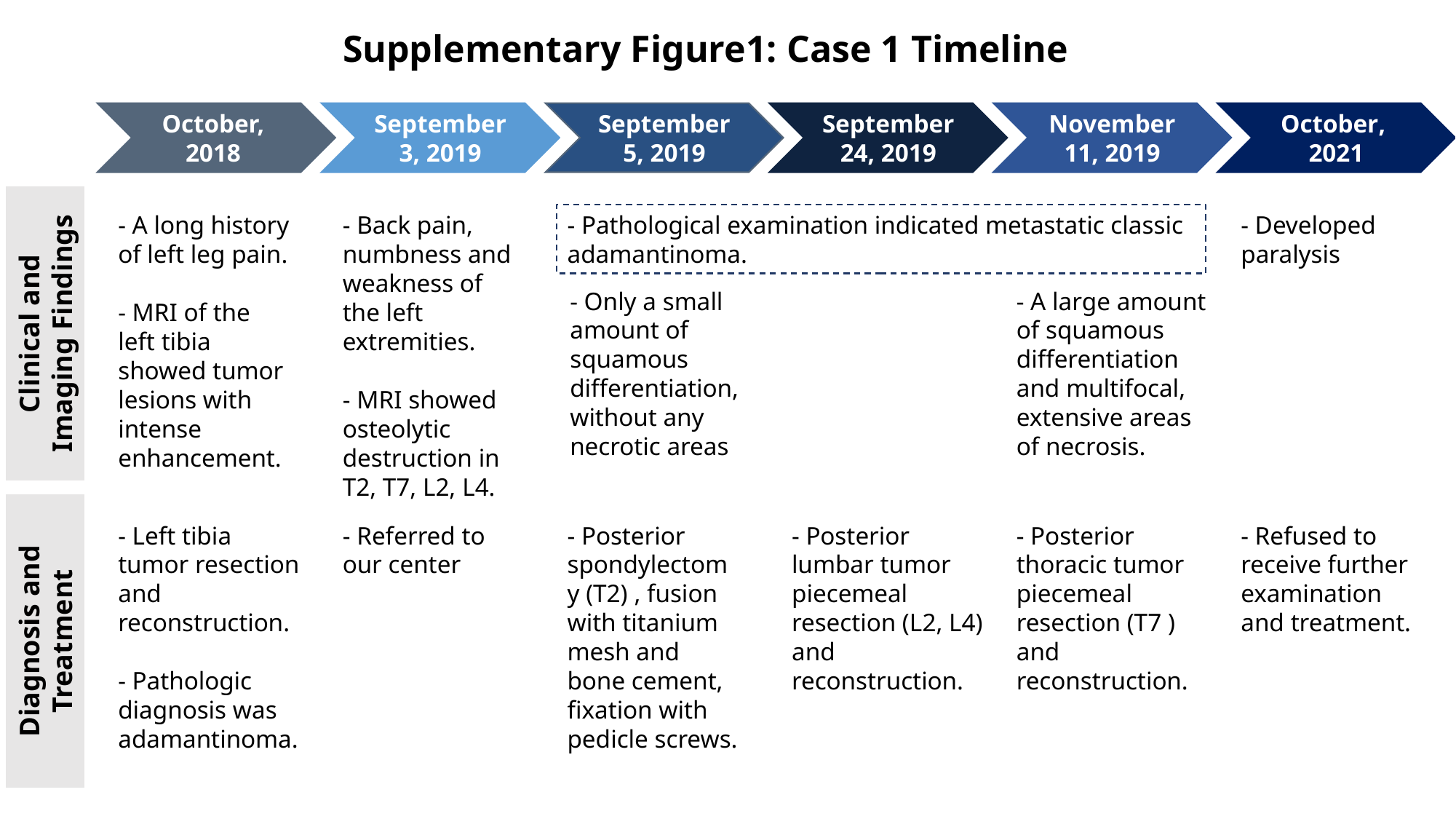

Supplementary Figure1: Case 1 Timeline
October,
2018
September 3, 2019
September 5, 2019
September 24, 2019
November 11, 2019
October,
2021
Clinical and Imaging Findings
- A long history of left leg pain.
- MRI of the left tibia showed tumor lesions with intense enhancement.
- Developed paralysis
- Back pain, numbness and weakness of the left extremities.
- MRI showed osteolytic destruction in T2, T7, L2, L4.
- Pathological examination indicated metastatic classic adamantinoma.
- Only a small amount of squamous differentiation, without any necrotic areas
- A large amount of squamous differentiation and multifocal, extensive areas of necrosis.
Diagnosis and Treatment
- Left tibia tumor resection and reconstruction.
- Pathologic diagnosis was adamantinoma.
- Posterior spondylectomy (T2) , fusion with titanium mesh and bone cement, fixation with pedicle screws.
- Refused to receive further examination and treatment.
- Referred to our center
- Posterior lumbar tumor piecemeal resection (L2, L4) and reconstruction.
- Posterior thoracic tumor piecemeal resection (T7 ) and reconstruction.

## Slide 2
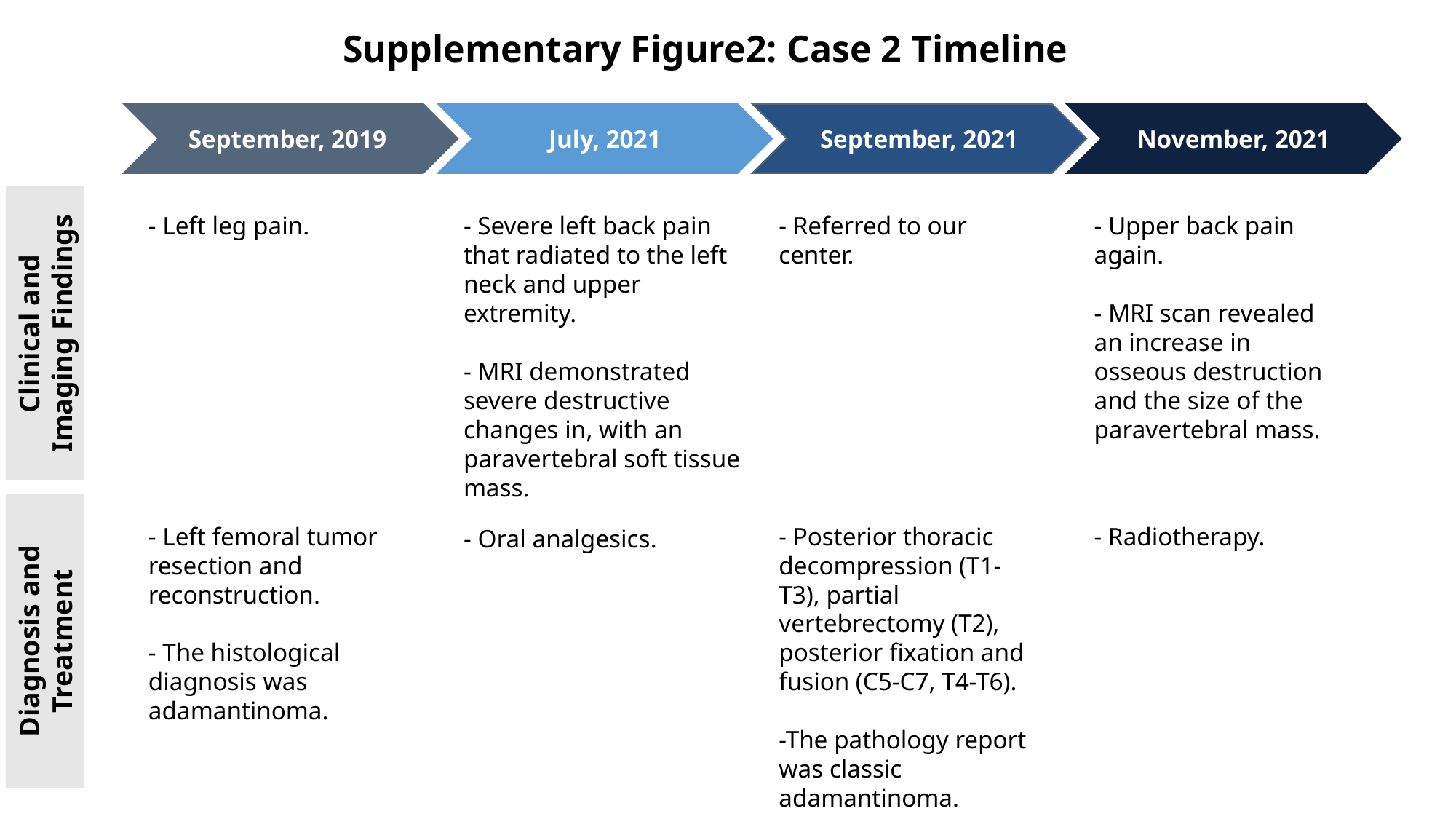

Supplementary Figure2: Case 2 Timeline
September, 2019
July, 2021
September, 2021
November, 2021
Clinical and Imaging Findings
- Left leg pain.
- Severe left back pain that radiated to the left neck and upper extremity.
- MRI demonstrated severe destructive changes in, with an paravertebral soft tissue mass.
- Referred to our center.
- Upper back pain again.
- MRI scan revealed an increase in osseous destruction and the size of the paravertebral mass.
Diagnosis and Treatment
- Left femoral tumor resection and reconstruction.
- The histological diagnosis was adamantinoma.
- Posterior thoracic decompression (T1-T3), partial vertebrectomy (T2), posterior fixation and fusion (C5-C7, T4-T6).
-The pathology report was classic adamantinoma.
- Radiotherapy.
- Oral analgesics.
